# Epidemiology of Vasomotor Rhinitis

**DOI:** 10.1097/WOX.0b013e3181ac91ae

**Published:** 2009-06-15

**Authors:** Russell A Settipane

**Affiliations:** 1 From the Allergy & Asthma Center, a clinical teaching site of the Warren Alpert School of Medicine at Brown University, Providence, RI , 95 Pitman Street, Providence, RI 02906

**Keywords:** vasomotor rhinitis, epidemiology, nonallergic rhinopathy, nonallergic rhinitis

## Abstract

Vasomotor rhinitis is the most common form of nonallergic rhinitis, comprising approximately 71% of all nonallergic rhinitis conditions. Although the epidemiology of this subtype of nonallergic rhinitis has not been definitively studied, it is estimated that 14 million Americans suffer from vasomotor rhinitis, with a worldwide prevalence approaching 320 million.

## Introduction

vasomotor rhinitis (VMR) is a subtype of nonallergic rhinitis (NAR) that is unrelated to allergy, infection, structural lesions, systemic disease, or drug abuse. Because it is, by definition, an idiopathic form of rhinitis, a diagnosis can be made only when all other forms of rhinitis have been excluded. Determination of the epidemiology of VMR is confounded by numerous challenges as shown in Table 1. The first challenge in determining the epidemiology of VMR is gaining agreement on the definition of this form of rhinopathy. As with other forms of NAR, VMR is characterized by periodic or perennial symptoms of rhinitis that are not due to IgE-dependent events. A number of consensus statements have put forth definitions of VMR, the most recent being the report of the 2008 AAAAI Joint Task Force on Practice Parameters, *The diagnosis and management of rhinitis: an updated practice parameter*, which defines VMR (idiopathic rhinitis) as a "heterogeneous group of patients with chronic nasal symptoms that are not immunologic or infectious in origin and are usually not associated with nasal eosinophilia."[1] The clinical characteristics of VMR are further elaborated in the article by Bernstein[3] in this issue.

**Table 1:** Challenges in Determining the Prevalence of Vasomotor Rhinitis

Agreement on VMR definition
Requirement to rule out all other forms of rhinitis
Requirement to rule out chronic rhinosinusitis
Skin testing or determination of serum-specific IgE is required
Local IgE production without systemic detection may be present (entopy)[2]
Sinus imaging is rarely assessed in large epidemiologic studies
Nasal cytology is rarely assessed in large epidemiologic studies

Because VMR is a diagnosis that essentially requires the exclusion of all other forms of rhinitis, an appropriately performed epidemiological study would require protocol incorporating a multitude of standardized tests to rule out all other forms of rhinitis. Appropriate testing might include those tests listed in Table 2. The article by Benniger et al,[5] which will appear in the next issue of this journal, describes the proposed inclusion and exclusion criteria for research studies in nonallergic rhinopathy, including VMR, as agreed upon by the participants of this roundtable meeting (see "Consensus Review and Definition of Non-allergic Rhinitis With a Focus on Vasomotor Rhinitis, Proposed To Be Known Henceforth as Nonallergic Rhinopathy: Part 1. Introduction" in this Review Series issue for the listing of participants). To date, no prospective epidemiologic studies have applied such a protocol to evaluate a large population of rhinitis patients. Consequently, current estimates regarding the prevalence of VMR are fairly crude.

**Table 2:** Diagnostic Tests to Exclude Other Forms of Rhinitis[4]

CT imaging of the paranasal sinuses
Assays for specific IgE sensitivity (a) Skin testing (b) Serum testing (c) Local (nasal) testing (entopy)[2]
Nasal cytology
Intranasal allergen challenge
Ingestion challenge (gustatory rhinitis)
Thyroid function testing

## Methods

A literature search was performed using the following terms: vasomotor rhinitis, nonallergic rhinitis, idiopathic rhinitis, nonallergic noninfectious rhinitis, prevalence, and epidemiology. On the basis of this search and pertinent review articles, the reported prevalence rates of NAR and subtypes were compiled and the prevalence of VMR was extrapolated.

## Results

### Relative prevalence rates of allergic rhinitis versus nonallergic rhinitis

Although no studies specifically designed to examine the epidemiology of NAR or VMR have been reported, 9 epidemiologic studies report data regarding the relative prevalence of NAR in comparison to that of AR (Table 3)[6-15]. Seven of the 9 studies employed skin testing with variable techniques (prick, intradermal, both, or undefined) to distinguish nonallergic rhinitis from allergic rhinitis. Studies that did not discount positive skin tests unsupported by history (all except Mullarkey et al) or that employed intradermal (or undefined) skin testing[6-9] are likely to have overestimated the prevalence of allergic rhinitis and underdiagnosed VMR[4]. Two of the studies used either history alone or ICD9 (*International Classification of Diseases, Ninth Revision*) data to diagnose VMR, both of which are not well-established for diagnostic purposes[12,14]. Because none of the studies assessed for the presence of local (nasal) IgE production, known as entopy, VMR may have been overdiagnosed in some cases[2].

**Table 3:** Relative Rhinitis Prevalence by Author: Allergic Versus Nonallergic

Author	Year	N	AR%	NAR%	NAR Defined
Mullarkey et al[6]	1980	142	48	52	No history of allergen exacerbation. Negative skin tests or <2 PSTs unsupported by history and an IgE level <50 U/mL
Enberg[7]	1989	128	64	36	Negative SPTs and IDs to 36 allergens
Togias[8]	1990	362	83	17	Negative skin tests
Leynaert et al[10]	1999	1142	75	25	Negative SPTs to 9 allergens
Settipane et al[9]	2001	975	77*	23	Negative skin tests
Mercer et al[12]	2002	278	78	22	Negative SPTs to 20 allergens
Bachert et al[13]	2006	743	75	24	History only
Mølgaard et al[14]	2007	1186	77	23	Negative SPTs to 10 allergens
Schatz et al[15] †	2008	47,894	71	29	ICD9 Classification
**Total**		**52,850**	**71**	**29**	

*Including 34% mixed.

†Subtotal without Schatz et al: 76% AR; 24% NAR.

Abbreviations: SPT, skin prick test; ID, intradermal; PST, positive skin test; ICD9, *International Classification of Diseases, Ninth Revision*.

Despite the fact that some of these studies were performed in allergy outpatient settings, which would be anticipated to skew the reported prevalence rates toward the diagnosis of AR, the findings are fairly consistent and independent of the setting performed. These 9 studies, when added in total, are heavily influenced by the enormity of the data from Schatz et al, but when analyzed independently of the Schatz data, they reveal a relative prevalence rate of 76% allergic and 24% nonallergic--closely approximating a 3:1 ratio.

Three studies were identified that attempted to systematically subtype NAR by performing testing that included, at a minimum, nasal examination, skin testing for sensitivity to specific aeroallergens, total IgE, nasal cytology, and sinus x-rays (Table 4)[6,7,16]. Each of these 3 studies has significant limitations. Symptoms were poorly characterized, irritant triggers were not captured, skin test techniques were variably defined, sinus imaging was limited to sinus x-rays (known to have limited value), and nasal examination data were not presented. However, each of these studies did include examination of nasal cytology (albeit with variable methodologies) in an attempt to screen out NARES or eosinophilic rhinosinustits.

**Table 4:** Tests Used to Characterize Nonallergic Rhinitis

Test	**Mullarkey et al**[6]	**Enberg**[7]	**Settipane and Klein**[16]
Nasal exam	+	+	+
Skin test	+	+	+
RAST	-	+	-
Total IgE	+	+ (partial)	+
CBC	+	-	+
ESR	+	-	-
TSH	-	-	+
Cytology	+	+	+
Sinus x-ray	+	+ (partial)	+

Abbreviations: RAST, radioallergosorbent test; CBC, complete blood count; ESR, erythrocyte sedimentation rate; TSH, thyroid stimulating hormone.**Relative Prevalence Rates of NAR Subtypes**

The data from these 3 studies, when combined, total 200 NAR subjects. VMR was identified as the most common subtype, making up 71% of NAR diagnoses, with nonallergic rhinitis with eosinophilia syndrome (NARES) making up the majority of the remaining diagnoses (Table 5). The definitions of the NAR subtypes, VMR and NARES, used in each of the 3 studies differed slightly. Sex and age demographic data suggest a 2:1 female-to-male ratio and a higher mean age (40 years old) for VMR subjects as compared with that of allergic rhinitis subjects.

**Table 5:** Prevalence of VMR in a Nonallergic Rhinitis Population by Study

Investigator	N (% Female)	Mean Age (Population)	Definition of VMR	VMR % (n, VMR/n, NAR)
Mullarkey et al[6]	73 (sex not reported)	37.5 (VMR); 25.1 (AR)	Nasal congestion and/or rhinorrhea persisting for ≥3 months, with no Hx of allergen exacerbation; negative skin tests or <2 positive skin tests unsupported by Hx; IgE <50 U/mL; <25% eosinophils	71% (52/73)
Enberg [7]	46 (74%)	40.5 (NAR)	Nasal Sx persisting for ≥1 year with no cause determined; negative skin test; <5% eosinophils on nasal smear	87% (40/46)
Settipane and Klein[14]	78 (58%)	42 (NAR)	Nasal congestion/rhinorrhea persisting for ≥3 months; negative skin tests; normal IgE; <5% eosinophils on nasal smear	61% (44/72)
**Total**	**191**	**40.5**		**71% (136/191)**

Abbreviations: Hx, history; Sx, symptom.

## Discussion

### Estimated prevalence of nonallergic rhinitis in the United States and worldwide

The data from rhinitis epidemiology studies suggest that the ratio of AR prevalence (pure and mixed combined) to that of pure NAR is 3:1. This ratio can be extrapolated to determine a conservative estimate of the prevalence of NAR in the United States based on established prevalence rates of AR. If the assumption is made that 20% of the population suffers from AR,[17] then on the basis of current population estimates for the United States of just more than 300 million,[18] the US prevalence of AR is 60 million people. Applying the 3:1 (AR/NAR) ratio, approximately 20 million Americans would be expected to suffer from NAR (or approximately 7% of the total population). Given a current world population of 6.75 billion,[18] similar extrapolation suggests that approximately 450 million people suffer from NAR worldwide. It is not known whether VMR is equally prevalent throughout the world and whether local weather (humidity), climate, air pollution, or genetic factors affect VMR prevalence.

### Estimated prevalence of VMR in the United States and worldwide

The studies by Mullarkey,[6] Enberg,[7] and Settipane[16] unanimously support VMR as the most common NAR subtype, making up approximately 71% of NAR diagnoses, with NARES making up the majority of the remaining NAR conditions. Applying the 71% frequency of VMR occurrence to the 20 million Americans who suffer from NAR, it would be estimated that VMR affects 14 million people in the United States. Applying the same frequency to the 450 million worldwide population suffering from NAR yields an estimate of a worldwide prevalence of VMR of 320 million.

### Further characterization of VMR

VMR is often described as being characterized by nonallergic symptom triggers, including weather (changes in temperature or relative humidity), alcohol, tobacco smoke, dusts, automotive emission fumes, nonspecific irritant stimuli such as chlorine, and odors such as bleach, perfume, or solvents[1]. Unfortunately, no epidemiologic data exist to further categorize VMR based on trigger type. Sex and age demographic data specific to VMR is limited, but can be extrapolated from NAR data, suggesting a female predominance and an older population for NAR than for AR[4-6,8,14]. However, the trend toward female predominance remains unproven; it is possible that a study selection bias may have resulted if, as suspected, more females than males entered studies because of an increased likelihood to seek rhinitis care.

## Conclusions

Data regarding the prevalence of rhinitis, regardless of the type, are difficult to interpret. Contributing to this challenge is the observation that most population surveys have flawed designs[1]. Because skin testing or determination of serum-specific IgE is infrequently assessed in large epidemiologic studies, allergic causation is often not accurately differentiated from nonallergic causation. However, on the basis of the data that has been reported, it is clear that VMR is, by far, the most common subtype of NAR with a significant burden of illness in the United States and worldwide.

## Note

Received grant/research support from GlaxoSmithKline (GSK), Sanofi-Aventis Pharmaceuticals, Meda Pharmaceuticals, and Alcon Laboratories. He is a consultant, or on an advisory board or the speakers bureau, for GlaxoSmithKline (GSK), Sanofi-Aventis Pharmaceuticals, and Alcon Laboratories.

Presented at a roundtable conference held in December 2008 in Washington, DC. The meeting was sponsored by the TREAT Foundation (Washington, DC) and supported through an unrestricted educational grant from Meda Pharmaceuticals. The funding company did not have any input into the development of the meeting or the series, and the company was not represented at the roundtable meeting.
